# Utility of a 3 mm Bipolar Instrument in Laparoscopic Renal Surgery Using the Senhance Robotic System

**DOI:** 10.7759/cureus.65694

**Published:** 2024-07-29

**Authors:** Go Kaneko, Suguru Shirotake, Masafumi Oyama, Isamu Koyama

**Affiliations:** 1 Urologic Oncology, Saitama Medical University International Medical Center, Hidaka, JPN; 2 Gastroenterological Surgery, Saitama Medical University International Medical Center, Hidaka, JPN

**Keywords:** robot-assisted surgery, maryland bipolar instrument, 3 mm, bipolar electrocautery, laparoscopic renal surgery, senhance, robot

## Abstract

We report our initial experience and the utility of 3 mm bipolar forceps in laparoscopic renal surgery using the Senhance robotic system. We performed laparoscopic nephroureterectomy for upper tract urothelial carcinoma in two patients: an 80-year-old female with a left renal pelvic tumor and an 80-year-old male with a right ureteral tumor. Both surgeries were successfully completed without conversion to conventional laparoscopic surgery or laparotomy. The console times for the procedures were 101 and 108 minutes, with estimated blood losses of 5 and 50 milliliters, respectively. The postoperative courses were uncomplicated, with histopathological examinations confirming high-grade urothelial carcinoma with negative surgical margins in both patients. The 3 mm Maryland bipolar instrument was able to grasp membranes with sufficient gentleness and precision. The relatively narrow diameter of the shaft posed a challenge in terms of shaft strength; however, it did not deflect even when it was used to lift the kidney, indicating sufficient robustness. When utilized in the cutting mode, the incision capacity of the 3 mm Maryland bipolar instrument was higher than that of the 5 mm instrument, which allowed for expedient and precise incision. Since only the tissue held by the forceps was incised, it was possible to perform a safe incision even in areas near blood vessels and other organs. Although the tip of the 3 mm Maryland instrument is more sharply pointed than that of the 5 mm instrument, no tissue damage was observed even when the 3 mm instrument was used for blunt dissection. Our initial results suggest that the 3 mm Maryland bipolar instrument is efficacious for performing laparoscopic renal surgery. The instrument may be suitable for a range of surgical procedures in laparoscopic renal surgery using the Senhance system. Further studies are necessary to establish the role and effectiveness of this instrument in broader clinical applications.

## Introduction

The Senhance robotic system (TransEnterix, Morrisville, NC, USA), initially introduced as the TELELAP Alf-X system in 2012, represents an advancement in telesurgical technology [1]. The robotic platform features up to four independently positionable and controllable robotic arms, which are operated by a surgeon seated at a console equipped with laparoscopic handles [2].

This system boasts several novel characteristics, including an eye-tracking camera control system and haptic feedback that provides a tactile dimension to the surgeon's experience. Moreover, operations can be performed in a comfortable seated position, eliminating the risk of neck strain. These characteristics are different from the da Vinci surgical system (Intuitive Surgical, Sunnyvale, CA, USA), which is the most popular robotic system. The Senhance system is compatible with standard trocars used in conventional laparoscopy. Most instruments in the Senhance system are reusable without limited time, significantly reducing maintenance costs. Therefore, the maintenance cost is lower than that for the da Vinci system. The Senhance system was approved for general surgery, gynecology, urology, and thoracic surgery by European regulators and the Food and Drug Administration in the United States of America in 2014 and 2017, respectively. Since its approval by the Japanese Ministry of Health, Labour and Welfare in 2019 for 98 different laparoscopic procedures across five surgical fields (general, colorectal, gynecologic, pediatric, and urological), its application has expanded. In 2022, insurance coverage was extended to include 19 thoracic and seven additional laparoscopic procedures.

In urology, the Senhance system has demonstrated versatility and efficacy in a range of procedures [3-10]. Our previous report highlighted the system's capability to perform laparoscopic radical nephrectomy for renal cell carcinoma [7]. This procedure benefits from the enhanced three-dimensional visualization and elimination of hand tremors, enabling the surgeon to operate with exceptional precision and finesse from a comfortable seated position. Furthermore, our institution has utilized the Senhance system for laparoscopic adrenalectomy for adrenal tumors and laparoscopic total nephroureterectomy for upper tract urothelial carcinoma (UC).

Recently, the system’s instrument range has expanded to include 3 mm instruments. While their effectiveness has been reported in gynecological, general surgical, and pediatric procedures [11-13], their utility in urological applications has not been verified. Herein, we present our initial experience with the use of a 3 mm bipolar instrument in laparoscopic renal surgery, with a discussion of potential benefits and effectiveness in urological procedures.

## Case presentation

Case 1

**Figure 1:**
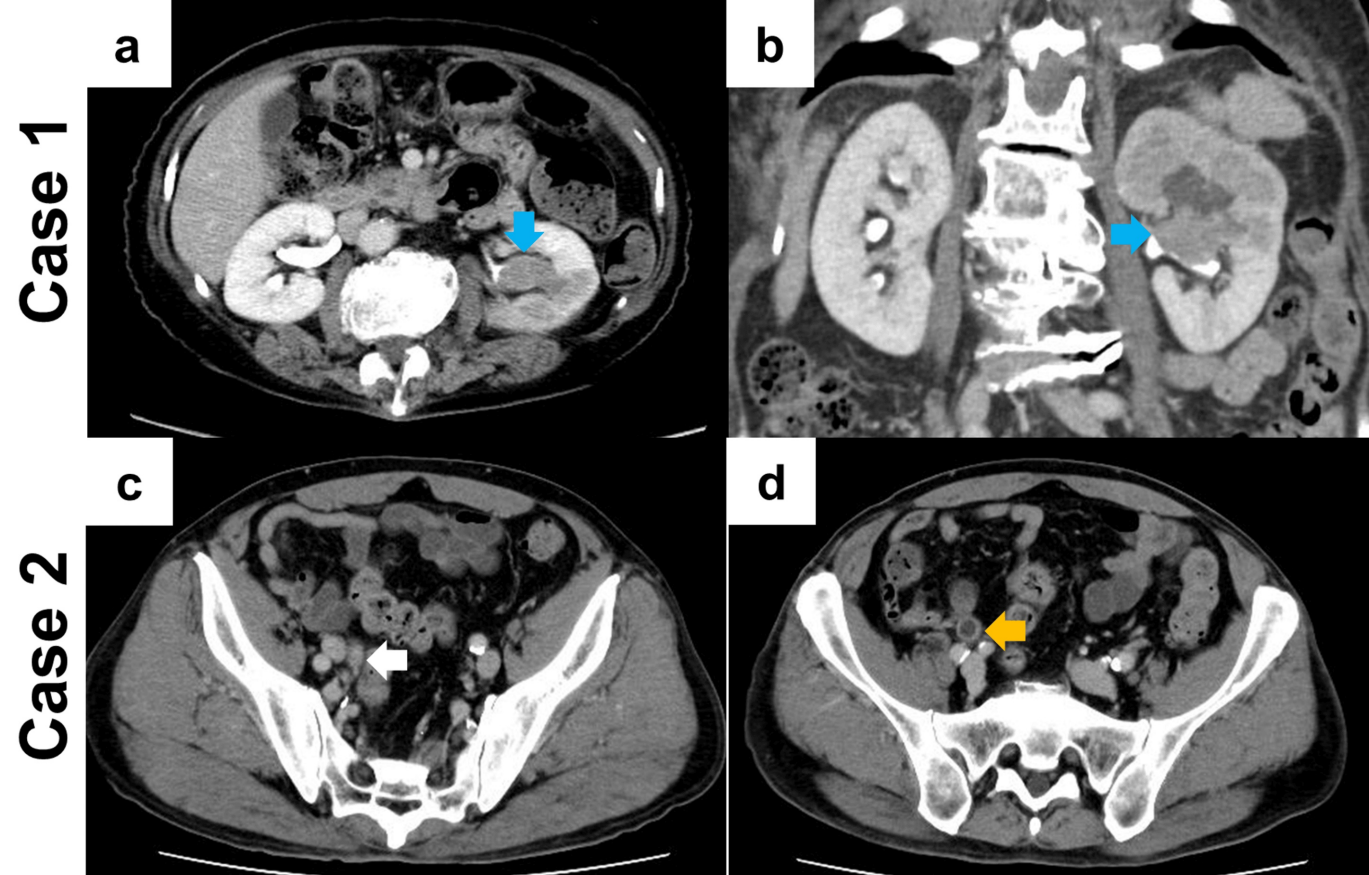
Computed tomography in Cases 1 and 2 (a and b): Computed tomography (CT) demonstrating a left renal pelvic tumor enhanced by contrast medium, with a diameter of 30 mm in Case 1. There is suspicion of parenchymal invasion (a: axial section, b: coronal section). The blue arrow indicates a left renal pelvic tumor (c and d): CT demonstrating a right ureteral tumor (white arrow) exhibiting contrast enhancement near the right iliac vessel and a thickened ureteral wall (orange arrow) on its cephalic side in Case 2.

An 80-year-old female (body mass index: 21.6 kg/m^2^) with a history of diabetes mellitus and pacemaker insertion for atrial fibrillation presented to her local physician with a complaint of gross hematuria for the past four months. Computed tomography (CT) revealed the presence of a left renal pelvic tumor, leading to a referral to our institution for further evaluation. Urine cytology demonstrated the presence of high-grade UC, as defined by the Paris system. CT showed a contrast-enhanced tumor within the left renal pelvis with a diameter of 30 mm (Figure 1a, 1b). The parenchymal invasion was suspected, and a diagnosis of left renal pelvis cancer (clinical T3N0M0) was made. Senhance-assisted laparoscopic nephroureterectomy was planned via an intraperitoneal approach.

Case 2

An 80-year-old male (body mass index: 25.3 kg/m^2^) who had undergone Bacillus Calmette-Guerin intravesical injection therapy for bladder carcinoma in situ one year earlier was referred to our hospital for further examination of a right ureteral tumor identified by CT by a neighboring hospital. Urine cytology demonstrated the presence of high-grade UC, as defined by the Paris system. CT revealed a ureteral tumor, which exhibited contrast enhancement near the right iliac vessel (Figure 1c) and a thickened ureteral wall on its cephalic side (Figure 1d). Ureteroscopy revealed a papillary tumor at the same site, which was biopsied and confirmed as high-grade UC. Senhance-assisted laparoscopic nephroureterectomy via an intraperitoneal approach was planned based on the diagnosis of right ureteral cancer (clinical T2N0M0).

Surgical technique

Case 1

**Figure 2:**
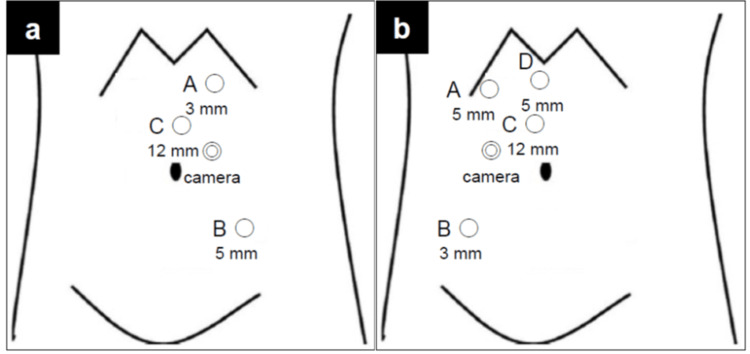
Trocar position a: Trocar position in the left laparoscopic nephroureterectomy using the Senhance system. The camera port is inserted at the exterior edge of the abdominal rectus muscle around the umbilicus. A 3 mm port is inserted at the exterior edge of the abdominal rectus muscle caudal to the arcus costalis (A). A 5 mm trocar is inserted in a lateral position relative to the exterior edge of the abdominal rectus muscle caudal to the camera port (B). Then, a 12 mm trocar is inserted around the umbilicus for the assistant (C). b: Trocar positioning in the right laparoscopic nephroureterectomy using the Senhance system The trocar positioning mirrored that performed in left laparoscopic nephroureterectomy using the Senhance system (camera port and A-C). Then, a 5 mm trocar is inserted at the epigastric area to elevate the liver (D).

**Figure 3:**
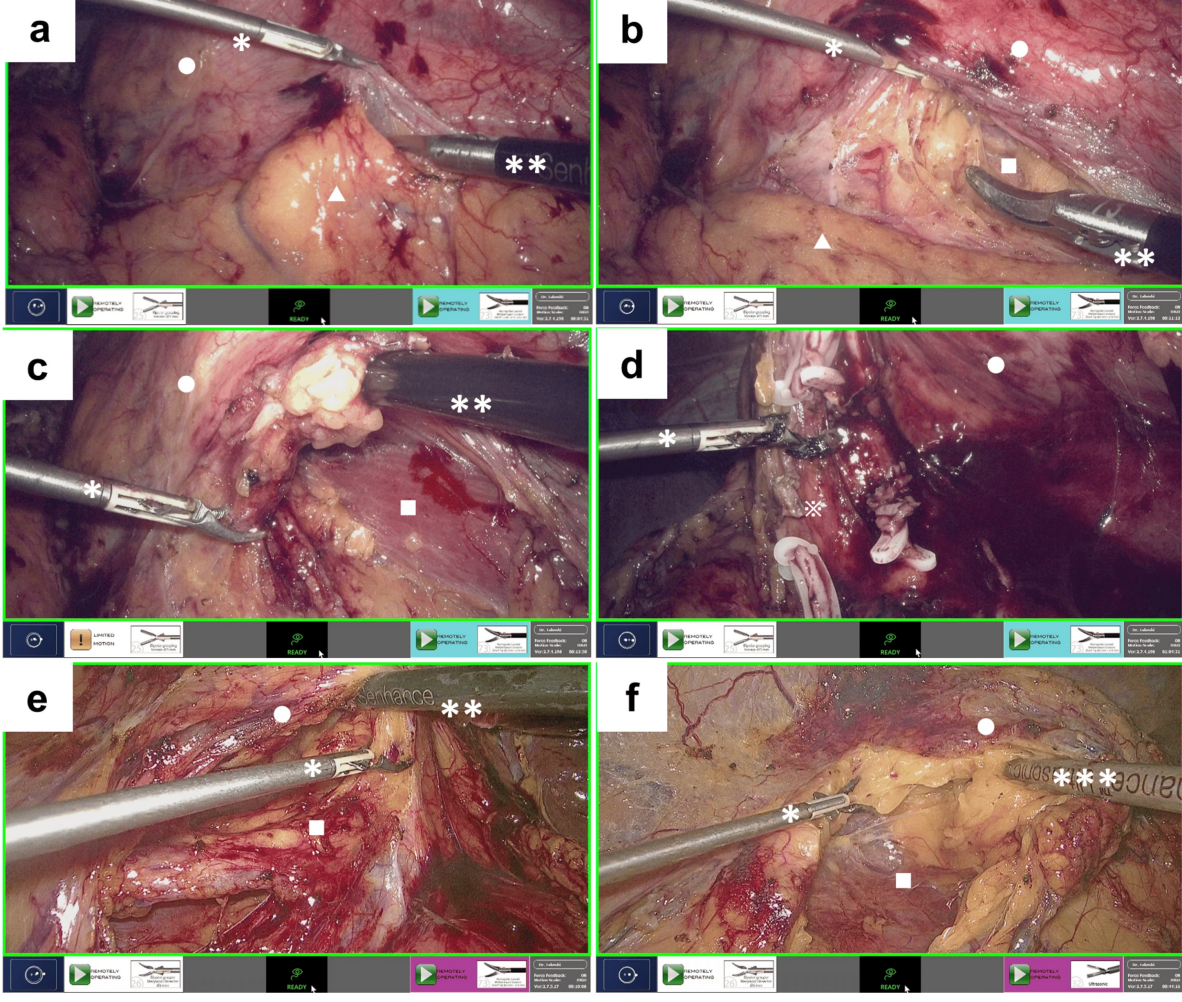
Intraoperative findings demonstrated the efficacy of the 3 mm Maryland bipolar instrument a: The 3 mm Maryland bipolar instrument is capable of delicately grasping the membrane, and the grasp is sufficiently robust to lift the kidney. b: The shaft of the 3 mm Maryland bipolar instrument does not exhibit any deflection when the kidney is elevated by the shaft. c and d: The psoas window (c) is precisely deployed, and the kidney and adrenal gland are efficiently dissected (d) using the 3 mm Maryland bipolar instrument, which is employed for cutting and coagulation. e and f: The psoas window (e) is efficiently deployed, and dissection around the kidney (f) is performed using the cutting and coagulation mode of the 3 mm Maryland bipolar instrument. *, **, ***, ○, △, and □ indicate the 3 mm Maryland bipolar instrument, 5 mm monopolar scissors, ultrasonic device, the kidney, the mesenterium, and the psoas muscle, respectively.

The patient was positioned in a right lateral position under general anesthesia, and a camera port was inserted at the exterior edge of the abdominal rectus muscle around the umbilicus (Figure 2a). Once the pneumoperitoneum had been established, three trocars were inserted (Figure 2a). The distance between ports A and B and the camera port were both 9 cm. A 10 mm rigid laparoscope (Karl Storz), 3 mm Maryland bipolar instrument, and 5 mm monopolar scissors were inserted via the camera port, a 3 mm trocar (port A), and a 5 mm trocar (port B), respectively, and connected to the Senhance system. A 12 mm trocar for the assistant was inserted in a position closer to the midline, situated between port A and the camera. A vessel-sealing device (Enseal, Ethicon, New Jersey, USA), a straight laparoscopic electrode (Endopath, Ethicon), and clips were utilized by the assistant for sharp dissection, suction, and the clamping of the renal artery and vein, respectively. These were performed from port C. The 3 mm Maryland bipolar instrument was connected to the VIO 3 electrosurgical unit (Erbe, Tübingen, Germany). The settings were configured to Autocut mode at 5.5 watts for incision and Soft Coag mode at 6 watts for coagulation. All procedures were conducted using the conventional approach for left laparoscopic nephroureterectomy. The kidney and upper ureter were dissected laparoscopically, and the lower ureter and bladder cuff were resected via laparotomy. The 3 mm Maryland bipolar instrument demonstrated the capacity to delicately grasp the membrane (Figure 3a). Moreover, the shaft did not exhibit any deflection when the kidney was elevated (Figure 3b). The deployment of the psoas window (Figure 3c) and dissection between the kidney and adrenal gland (Figure 3d) were facilitated by the 3 mm Maryland bipolar instrument, which was employed for cutting and coagulation. This approach enabled the execution of a precise dissection while simultaneously maintaining control of the bleeding.

Case 2

The patient was positioned in a left lateral decubitus position under general anesthesia, with trocar placement mirroring described in Case 1 (Figure 2b). Then, a 5 mm trocar was inserted into the epigastric area to elevate the liver (port D). A rigid laparoscope, 5 mm monopolar scissors, and 3 mm Maryland bipolar instrument were inserted from the camera port, a 5 mm trocar (port A), and a 3 mm trocar (port B), respectively, and docked to the Senhance system. The assistant provided support from a 12 mm port (port C). All procedures were conducted using conventional right laparoscopic nephroureterectomy. The cutting and coagulation of the 3 mm Maryland bipolar instrument were effective for the deployment of the psoas window (Figure 3e) and the dissection around the kidney (Figure 3f).

Peri- and postoperative results

In both cases, the procedure was successfully completed without conversion to conventional laparoscopic nephroureterectomy or laparotomy. The console time and estimated blood loss in Case 1 were 101 minutes and 5 milliliters, and in Case 2, were 108 minutes and 50 milliliters, respectively. The postoperative course was uncomplicated. The histopathological diagnosis was high-grade UC (pathological T3) in Case 1 and high-grade UC (pathological T3) with lymphovascular invasion in Case 2. In both cases, the surgical margins were negative.

## Discussion

We described our initial experience with a 3 mm Maryland bipolar instrument in laparoscopic renal surgery using the Senhance system. We found that it was able to grasp membranes with sufficient gentleness and precision. The relatively narrow diameter of the shaft presented a challenge in terms of shaft strength; however, the shaft did not deflect even when used to lift the kidney, indicating sufficient robustness. The utilization of a 3 mm Maryland bipolar instrument in both cutting and coagulation modes during the dissection around the kidney permitted the precise dissection of the tissue with controlled bleeding.

In our previous report [7], we primarily utilized 5 mm monopolar scissors in the right hand for coagulation and cutting and 5 mm bipolar forceps in the left hand for coagulation in laparoscopic renal surgery using the Senhance system. The only energy device that made incisions was monopolar scissors. However, we encountered a situation where the operation could not be performed smoothly due to concerns about injury when using monopolar scissors for incision in cutting or coagulation mode around vascular and other organs. Laparoscopic renal surgery using the Senhance system may be difficult to complete in certain cases such as those with severe adhesions around the kidney. In addition to its use in coagulation mode, the 5 mm bipolar instrument was employed in cutting mode, with various settings for the electrocautery being tried. However, the incision capacity was insufficient to allow for accurate and safe incision, and no solution was found.

In the present cases, we attempted to substitute the 5 mm forceps operated by the left hand with a 3 mm Maryland bipolar instrument. When utilized in the cutting mode, the incision capacity was considerably higher than that of the 5 mm instrument, allowing for expedient and precise incision. Furthermore, since only the tissue held by the forceps was incised, it was possible to perform a safe incision even in areas near blood vessels and other organs. Although the tip of the 3 mm instrument was more sharply pointed than that of the 5 mm instrument, no tissue damage was observed even when the 3 mm instrument was used for blunt dissection.

In recent years, the use of the "double bipolar method," in which both hands operate the bipolar instrument during robotic surgery using the da Vinci robotic system (Intuitive Surgical Inc., Sunnyvale, CA, USA), has become widespread in the fields of general surgery and gynecology in Japan [14-17]. We previously reported the effectiveness of the double bipolar method in partial cystectomy using the da Vinci system [18]. In this procedure, the Maryland bipolar instrument is usually controlled by the surgeon’s dominant hand and other bipolar instruments by the non-dominant hand. Cutting and coagulation vary depending on how the tissue is grasped with the Maryland bipolar instrument. When used with a thick bite, the Maryland bipolar instrument can coagulate the grasped tissue; on the other hand, when used with a thin bite, the tissue can be cut with a spark. To the best of our knowledge, there are no published reports of the use of a bipolar instrument in cutting mode in the Senhance system in the urological field. In the present cases, the electrocautery cutting mode and coagulation mode were set separately, rather than the coagulation and incision modes being used according to the tissue-grasping technique. Although both incisional and coagulation performances were satisfactory, further experimentation with various settings is necessary in order to establish the optimal setting.

At present, the 5 mm monopolar scissors are used with the right hand, primarily for blunt dissection and coagulation hemostasis. Our findings suggest that the 3 mm Maryland bipolar instrument can be used safely and effectively for blunt dissection and exhibits high hemostatic performance for soft coagulation. In the future, we plan to commence laparoscopic renal surgery with the Senhance system utilizing the double bipolar method with 3 mm Maryland bipolar instruments in both hands.

## Conclusions

The 3 mm Maryland bipolar instrument in the Senhance system was useful in a variety of situations including for tissue grasping, blunt dissection, incision, and coagulation. Our findings suggest that the 3 mm Maryland bipolar instrument is useful in the more widespread utilization of laparoscopic renal surgery using the Senhance system. In addition, we intend to investigate the potential applications of the 3 mm Maryland bipolar instrument in urologic laparoscopic surgery other than laparoscopic renal surgery.
